# Intracellular Delivery of Proteins with Cell-Penetrating Peptides for Therapeutic Uses in Human Disease

**DOI:** 10.3390/ijms17020263

**Published:** 2016-02-22

**Authors:** Ana Dinca, Wei-Ming Chien, Michael T. Chin

**Affiliations:** 1 Department of Pathology, University of Washington, Seattle, WA 98109, USA; adinca@uw.edu; 2 Department of Medicine, Division of Cardiology, University of Washington, Seattle, WA 98109, USA; chienw@u.washington.edu

**Keywords:** enzyme replacement therapy (ERT), cell-penetrating peptides (CPPs), enzyme deficiency, protein therapeutics

## Abstract

Protein therapy exhibits several advantages over small molecule drugs and is increasingly being developed for the treatment of disorders ranging from single enzyme deficiencies to cancer. Cell-penetrating peptides (CPPs), a group of small peptides capable of promoting transport of molecular cargo across the plasma membrane, have become important tools in promoting the cellular uptake of exogenously delivered proteins. Although the molecular mechanisms of uptake are not firmly established, CPPs have been empirically shown to promote uptake of various molecules, including large proteins over 100 kiloDaltons (kDa). Recombinant proteins that include a CPP tag to promote intracellular delivery show promise as therapeutic agents with encouraging success rates in both animal and human trials. This review highlights recent advances in protein-CPP therapy and discusses optimization strategies and potential detrimental effects.

## 1. Introduction

Intracellular delivery is crucial for many therapeutic molecules with targets inside the cell; however, the ability to get large molecules across the plasma membrane poses great challenges. Owing to reduced bioavailability *in vivo*, many promising candidates are not developed to their full therapeutic potential [1]. Thus, the need to find effective methods that enable the delivery of therapeutic compounds across the plasma membrane is urgent. Cell-penetrating peptides (CPPs), also referred to as protein-transduction domains (PTDs), have been widely used to facilitate the entry of proteins, small molecules, DNA and RNA into cells (reviewed in [2]). The first CPP was discovered serendipitously in the late 1980s when a group studying the human immunodeficiency virus (HIV) described the PTD of the trans-activator of transcription protein, Tat [3]. In the ensuing decades, CPPs attracted a great deal of interest for their potential in both biology research and therapy. They consist of short sequences of 8 to 30 amino acids in length that can facilitate entry of a variety of molecules into cells, including nucleic acids, proteins and synthetic drugs, usually with limited cell specificity. While multiple naturally derived sequences have been identified, a number of chimeric and synthetically designed peptides have also been generated, aiming to improve cellular uptake and provide more cellular and sub-cellular specificity. The peptides are usually classified according to their physical and chemical properties into cationic and amphipathic, or metabotropic, categories. The first two CPPs to be discovered, Tat and penetratin (a peptide derived from the drosophila homeobox protein, antennapedia, also referred to as Antp [4]), are highly cationic peptides [5]. Later work led to the discovery of peptides that carry a lower charge and are more hydrophobic in nature [6].

The ability of CPPs to deliver large cargo to the intracellular environment provides an opportunity to deliver intracellular, therapeutically active proteins, thus providing a novel platform for the development of medical treatments using molecules that had thus far been considered improbable for therapy. This review will focus on the potential therapeutic uses of CPPs in promoting the uptake of biologically active proteins important in both inherited or acquired forms of protein deficiency. We will also describe the barriers that must be overcome before these CPP-protein therapies can become therapeutically useful.

## 2. Mechanisms of Intracellular Delivery

The mechanisms by which CPPs promote uptake of associated molecules are influenced by the physico-chemical properties of the different penetrating peptides, the associated molecular cargo, and the variety of cells and tissues being treated. Two general models of uptake have been proposed: energy-independent direct translocation across the plasma membrane to the cytosolic compartment [7,8] and endocytosis [9,10]. Several types of endocytosis have been demonstrated for different peptides, including caveolar, clathrin-mediated and macropinocytosis [11,12,13]. In the case of endocytosis, the fate of the CPP-cargo complexes depends on their ability to escape the endosomal compartment before being delivered to lysosomes for degradation. It is important to note that this mechanism of cellular delivery can limit the effectiveness of CPP-based deliveries by decreasing the amount of cargo that is ultimately active and correctly targeted [14]. Results from studies conducted thus far, however, suggest that the mechanism of uptake is highly dependent on the type of cargo, making it difficult to predict which penetrating peptide should be selected in each case [15,16]. Strategies for increasing endosomolysis, thereby making more active cargo available, involve the use of pH-responsive elements, which are active in the acidic environment of endosomes without disrupting other organellar membranes. These include the addition of a short peptide derivative of the influenza virus protein hemagglutinin (INF) or the use of a synthetically designed peptide, GALA [17,18].

Mechanisms of CPP-mediated cellular uptake have been discussed extensively in previous reviews, including an article on membranotropic peptides in the current CPP special issue, to which the reader is referred for more extensive detail [6,15,19]. In addition, CPPsite [20], an online database of CPPs, describes the more than 1,800 known CPPs and provides information about uptake mechanisms for each individual peptide.

## 3. Cell-Penetrating Peptides (CPPs) Facilitate Intracellular Protein Therapy

The availability of CPPs to deliver therapeutically active cargo to the intracellular environment provides a novel platform for the development of medical treatments using molecules that would otherwise be ineffective without cellular entry. Proteins used as potential therapeutic molecules have traditionally been limited to those that function at the cell surface through ligand-receptor interaction, such as insulin, growth hormone, interferon, *etc.* Proteins that function intracellularly, however, may also be used as therapeutic molecules if cellular uptake of an intact, enzymatically active form can be achieved. Conjugation of cell-penetrating peptides to these proteins or even concurrent delivery of CPPs with other proteins can potentially circumvent this issue, thus facilitating the development of novel therapies. This review focuses on advancements made in CPP-aided protein therapies as new tools for medicine.

### 3.1. Systemic Delivery of CPP-Tagged Protein Therapeutics in Vivo

Therapeutic proteins can be delivered enterally or parenterally through intravenous, subcutaneous or inhaled routes of entry. Proteins must traverse mucosal barriers in some cases, travel through the extracellular space into the bloodstream, before entering target cells. Conjugation of CPPs to biotherapeutics or co-administration of CPPs can resolve some of the commonly encountered problems with systemic routes, by increasing cellular absorption and ensuring bioavailability to otherwise impenetrable organs, such as the brain [21].

#### 3.1.1. Facilitated Intranasal and Intestinal Delivery of Insulin

Insulin is a circulating polypeptide hormone that acts at the surface of the cell through binding to its receptor tyrosine kinase to promote the uptake of glucose into the cell. In diabetes mellitus, insulin deficiency or insulin resistance is often treated with administration of subcutaneous insulin, which is then absorbed into the bloodstream and circulates to target tissues. To maintain circulating glucose homeostasis, insulin must usually be administered subcutaneously several times a day for prolonged periods, which can lead to patient discomfort. Nasal co-administration of insulin and penetratin in rats leads to 50% bioavailability and significant lowering of blood glucose, while intranasal administration of insulin alone does not lead to a decrease in blood glucose concentration [22]. The presumed mechanism is by transport across the nasal epithelial cells with subsequent release into the systemic circulation. A later study by the same group examined the potential of oral administration of insulin and an enzyme-resistant d-form of penetratin to facilitate intestinal uptake after oral administration. The oral route is the least invasive method of administration and would more closely mimic endogenous spikes of insulin seen in healthy people. Orally administered peptides are subject to enzymatic degradation in the intestinal fluid and the authors addressed this by examining the resistance to degradation of l- and d-forms of penetratin, as well as insulin, in intestinal fluid. While both forms of penetratin decreased the degradation rate for insulin, d-penetratin showed an increased resistance compared to l-penetratin and led to the highest amount of bioavailability, which in this case was limited to 18% [23].

#### 3.1.2. Enhancement of Cell-Mediated Immunity and Facilitation of Anticancer Therapy

Therapeutic protein and peptide-based vaccines typically induce a humoral antibody response but not a cell-mediated immune response, based on antigen processing through primarily the endocytic pathway and presentation via the MHC class II pathway. Activation of the cytotoxic T-cell lymphocyte (CTL) response requires antigen processing through the MHC class I pathway. CPPs have been shown to promote cytosolic uptake of vaccine antigens, resulting in cross-presentation by the MHC I pathway, thereby enhancing the cytolytic response. For example, a non-covalently bound cell-penetrating peptide, LAH4, facilitated the cellular entry of a tyrosinase-related protein 2 (TRP2) peptide vaccine, resulting in enhanced cross-presentation to CTLs, leading to antitumor effects against TRP2-expressing tumors in a mouse model [24].

Autoantibodies against double-stranded DNA can mediate cell penetration and target to the nucleus without pathological effects on the cell. A single chain variable fragment (scFv) from one such antibody, 3E10, can facilitate cell entry of therapeutic proteins and has been used to construct bispecific antibodies targeting cancer cell growth by enhancing p53 tumor suppressor function [25]. Mutations in the gene-encoding p53 have been implicated in the majority of human cancers [26], and its tumor-suppressor function is suppressed by a protein-protein interaction with murine double minute 2 homolog (MDM2). A monoclonal antibody directed against MDM2, 3G5, prevents MDM2 from binding and inhibiting p53 [27]. 3G5 is not able to enter cells, but linking its variable fragment to that of the cell-penetrating 3E10 creates a bispecific antibody that can successfully inhibit MDM2, leading to cell growth arrest *in vitro* and reduction of tumor growth *in vivo* [28].

Another compound aimed at preventing p53 degradation in tumors is a novel amphipathic CPP, p28. Originally derived from amino acids 50−77 of azurin, a redox protein from the pathogen *Pseudomonas Aeruginosa*, p28 demonstrates protein transduction domain capabilities with an affinity for cancer cells and antitumor effects. P28 can promote uptake of heterologous proteins such as green fluorescent protein and glutathione *S*-transferase into cultured cells [29]. The actual protein transduction domain consists of amino acids 50−67, designated p18, while amino acids 68−77 are at least partially responsible for the antitumor effects [30]. p28 binds and stabilizes p53, exerting anticancer effects through p53-mediated apoptosis [31]. After demonstrating efficacy in preclinical trials, p28 entered clinical trials in humans, where it has shown strong evidence of anti-tumor activity and improved survival in patients [32]. Because p28 also has anti-angiogenic effects on endothelial cells, it can play a dual role in inhibiting tumor growth [33]. This is an important proof-of-concept study demonstrating that protein transduction domains can be coupled with other peptides and used clinically in cancer therapy.

#### 3.1.3. Enzyme Replacement Therapy

A class of emerging therapeutics introduces exogenous enzymes into cells deficient in those proteins. Enzyme replacement therapy (ERT) is commercially available for various lysosomal storage diseases (LSDs) and represents a potentially exciting avenue for other inherited enzyme deficiency disorders that currently lack available treatments. Enzyme replacement therapies have the benefit of well-understood safety profiles when compared with gene therapies and also generally show increased specificity compared with small molecule mimetics [34]. ERTs currently in development for various inherited disorders are described in the following paragraphs.

In neutrophils, an intact phagocyte NADPH oxidase (PHOX) complex is required to produce reactive oxygen species (ROS) that facilitate clearing of bacterial infections by promoting destruction of phagocytized bacteria. In chronic granulomatous disease (CGD), phagocytic cells are unable to destroy internalized bacteria efficiently due to mutations in one of the four essential subunits of the PHOX complex. Honda and colleagues restored the ability to produce ROS in CGD patient-derived neutrophils by transducing them with one of two of the essential subunits of the PHOX complex (p47 or p67) linked to the PTD Hph-1. These proteins are normally localized in the cytosol and translocate to the plasma membrane, the site of ROS production, only upon neutrophil activation. The exogenously delivered proteins localized in the cytosol in control cells and did not produce excess ROS. These recombinant enzymes only translocated to the membrane after neutrophils were activated with phorbol myristate acetate treatment, and subsequently restored the ability of the neutrophils to produce ROS [35]*.*

Lipoamide dehydrogenase (LAD, also known as E3) deficiency is an inherited mitochondrial disorder with a variable clinical course resulting in severe metabolic disturbances. The LAD protein is part of several large complexes in the mitochondrial matrix responsible for the metabolism of amino acids and carbohydrates. Lorberboum and colleagues reported the successful delivery of two different Tat-coupled mitochondrial enzymes, LAD and nicotinamide adenine dinucleotide dehydrogenase complex I assembly factor (NDUFAF4) to mice and patient cells, respectively [36,37]. Coupling the LAD enzyme with a Tat-penetrating peptide facilitated the delivery of highly purified recombinant protein to both patient-derived cells and mice deficient in LAD. The Tat peptide promoted uptake of LAD into cells and tissue and resulted in a measurable increase in LAD activity within 30 min and up to 48 h in mouse tissues. The increase in LAD activity could be correlated with an even higher increase in activity for one of the complexes that incorporates this enzyme, the pyruvate dehydrogenase complex (PDHC). These data suggest that only a small amount of enzyme replacement can result in correction of the deficit in downstream pathways. Mitochondrial complex I deficiency is a disorder caused by mutations in NDUFAF4, a protein involved in the assembly of complex I; the clinical manifestations range from perinatal lethality to adult-onset neurodegeneration. A recombinant protein containing the wild-type version of NDUFAF4 and the cell-penetrating peptide Tat is efficiently taken up by patient-derived NDUFAF4-deficient cells, resulting in a significant increase in complex I activity and improved mitochondrial function. Another inherited mitochondrial disorder with no available treatment is Friedrich’s ataxia. Recently, Vyas *et al.* have reported on efforts to develop a frataxin-Tat enzyme replacement therapy with encouraging results in mice [38].

X-linked myotubular myopathy (XLMTM) is a rare form of congenital myopathy resulting from mutations in the lipid phosphatase myotubularin. Lawlor and co-workers showed that a small amount of exogenous myotubularin linked to the 3E10Fv PTD identified by Weisbart [25], delivered intramuscularly, could significantly improve both local and distant muscle performance in a mouse model of XLMTM [39]. This enzyme plays a role in multiple cellular processes, including excitation-contraction coupling and apoptosis. Four intramuscular injections delivered over two weeks resulted in improvement in several parameters used to assess XLMTM, including excitation-contraction coupling and locomotion. The CPP used was thought to facilitate both local and systemic delivery when given intramuscularly.

CPPs have also been used as adjuncts to gene therapy in LSDs. LSDs are a group of inherited metabolic disorders arising from mutations that result in lysosomal accumulation of metabolic intermediates. Current treatment for these disorders consists of enzyme replacement by delivering the missing enzymes, tagged with mannose-6-phosphate, which promotes uptake and delivery to lysosomes mediated by the mannose-6-phosphate receptor [40]. More recently, gene therapy has been attempted in mouse models of various LSDs; however, delivering genes to cells via a viral vector can result in uneven distribution among cells. To circumvent this shortcoming in a mouse model of Fabry disease, Higuchi *et al.* included a Tat CPP downstream of the gene encoding the missing enzyme, α-Galactosidase A, demonstrating that the recombinant α-Gal A-Tat enzyme is able to diffuse to neighboring cells that may not have been transduced by the virus [41]. Neonatal Fabry mice that were injected with virus-encoding α-Gal A-Tat showed reduced accumulation of glycosphingolipids, a hallmark of the disease, compared to mice injected with virus-encoding wild-type α-Gal A. This study accentuates the complementary nature of protein engineering with CPPs and gene therapy.

CPP-mediated enzyme replenishment can also be used in non-inherited conditions. Reduced mitochondrial complex I activity has been linked to ischemia/reperfusion-induced damage after myocardial infarction [42]. In a rat model of ischemia/reperfusion, Tat-mediated administration of Nicotinamide adenine dinucleotide quinone internal oxidoreductase (Ndi1), the single-subunit yeast analog of complex I, demonstrated significant cardioprotective effects [43]. Ndi1 introduced into mammalian cells has been shown to work in parallel with endogenous complex I to provide electrons to ubiquinone. Intraperitoneal (IP) delivery of TAT-Ndi1 two h prior to ischemia/reperfusion resulted in localization of the recombinant protein to the inner mitochondrial membrane and subsequent reduction in infarct size by almost 50%. Even when TAT-Ndi1 was delivered at the onset of reperfusion in Langendorff-perfused hearts, it demonstrated protection against injury [44]. These experiments support TAT-Ndi1 as a promising therapeutic agent for reducing ischemia-reperfusion injury and possibly other disorders involving complex I dysfunction, such as mutations in one of the subunits of the enzyme.

### 3.2. Local Delivery

Systemic delivery of therapeutic proteins may in some situations be undesirable, such as when organ-specific deficiencies need to be addressed and also when potential therapies have toxic or off-target effects in certain tissues. CPPs that target specific tissues would circumvent these issues and have been reported for the heart [45], although most CPPs do not target specific tissues. Another option is to develop local delivery methods for CPP-conjugated proteins. Our group has recently developed methods for localized delivery of CPP-tagged Cre protein to heart and skeletal muscle in mice and have demonstrated successful DNA recombination in the targeted tissues [46]. In our proof-of-concept study, we used a CPP-tagged Cre protein, which we delivered by microinjection to either skeletal muscle or, via ultrasound guidance, to the heart of living animals. Two weeks post-injection, we found evidence of recombination in skeletal and cardiac myocytes, verifying the success of this targeted method. A similar approach can be used with other CPP-tagged proteins, in those cases where protein therapy can prove too toxic for systemic administration.

## 4. Benefits and Challenges of Utilizing Recombinant CPP-Tagged Proteins for Therapy

Traditionally, drug development approaches have not favored proteins as therapeutics, due to the difficulty of delivering them into cells, focusing instead on mimetic small molecule drugs [1]. While small molecules are better able to penetrate the cell membrane, they lack both the specificity and efficacy of peptides and proteins. Conjugation of therapeutic proteins and peptides to CPPs is an approach that shows great promise for developing novel therapies, but there are still challenges to overcome.

### 4.1. Tissue-Specific Targeting of Protein-CPP Therapeutics

One major challenge in the use of proteins for therapy is delivering them to the appropriate site of action; this challenge is twofold—getting the protein to the target tissue/cells and ensuring localization of the exogenous protein to the correct extra- or intracellular compartment. CPPs have been developed that reportedly deliver cargo to certain cell types/tissues, either by using CPPs which target specific cells or by taking advantage of unique features of the target microenvironment. Two different CPPs, p28 and NGR, have been reported to preferentially target cancer cells and tumor vasculature, respectively [30,47]. Several other cell-specific CPPs have been identified by phage display, including cardiac cells [45], endothelial cells [48] and dendritic cells [49], although these studies await further confirmation.

Attachment of a CPP could impact its function in live cells. It has been proposed that the presence of CPPs acidifies the environment in endosomes, thus facilitating escape. This same effect could also disrupt microdomains or organellar environments where a particular enzyme localizes. In addition, depending on where the CPP is conjugated to the protein, it could also prevent membrane-embedded enzymes from attaching to the membrane. A system in which the CPP is cleaved off once the protein reaches the intracellular environment would theoretically circumvent these issues. Efforts to develop cleavable CPPs, however, have thus far been limited.

Although coupling of an exogenous enzyme with a CPP can promote its entry into cells, endogenous targeting signals are usually relied upon for further trafficking to its subcellular compartment. One consequence of attaching polycationic CPPs to proteins is their propensity to direct localization to the nucleus; if the therapeutic enzyme is destined for a different subcellular location, then attachment of a CPP could interfere with its proper localization. Development of a class of synthetic mitochondrial and other organelle-specific targeting peptides is a potential solution for this problem [50,51,52,53].

### 4.2. Targeting to Specific Subcellular Compartments

Horton *et al.* have described a class of peptides comprised of both synthetic and natural residues known as mitochondria-penetrating peptides (MPPs) [50]. These peptides reportedly facilitate cellular uptake to levels comparable with cationic CPPs and in addition target the cargo to mitochondria. While there are no reports yet on combining protein therapy with these peptides, they have been successfully used to deliver the antibacterial methotrexate (Mtx) to mitochondria of human cells, thereby reducing the toxicity associated with the typical cytoplasmic delivery of this compound [54]. Due to similarities between mitochondrial and bacterial membranes, targeting of antimicrobials to this organelle allows for effective penetration of bacteria, leading to increased accumulation compared to non-targeted drug. In human cells, the MPPs lead to mitochondrial sequestration of the antimicrobial, which reduces host cell toxicity by preventing interaction with homologous targets.

More recently, a dual role antioxidant and mitochondria-penetrating peptide, mtCPP-1, was developed based on the Szeto-Schiller (SS) tetrapeptide antioxidants [52,55]. Like the SS peptides, mtCPP-1 alternates aromatic and basic residues, known to be important for targeting to the mitochondria. mtCPP-1 was able to successfully transport 5(6)-carboxyfluorescein (5-FAM) across the cell membrane and preferentially target it to the mitochondria. It was also able to reduce superoxide production in cultured cells treated with antimycin A more efficaciously than the SS peptide.

Another approach focuses on combining existing CPP motifs with mitochondrial-targeting sequences (MTS) to promote cellular entry and targeting to the mitochondria. Lin *et al.* based their dual peptide design on the MTS of the mitochondrially destined protein, aldehyde dehydrogenase, and the intracellular targeting properties of polyarginine peptides [53]. The combination of these two motifs results in cellular uptake and mitochondrial targeting of the non-cell-penetrating dye, 5-FAM. Surprisingly, the combination of MTS alone with dye leads to a non-negligible amount of uptake, presumably due to the amphipathic nature of the mitochondria-targeting peptide. Using the same technique of combining an MTS, in this case from the superoxide dismutase 2, with a polyarginine CPP, another group was able to show preferential delivery to mitochondria of an active protein, the mitochondrial transcription factor A (TFAM) [56,57]. TFAM is involved in mitochondrial DNA (mtDNA) replication and is required during development. A number of mitochondrial disease states result in decreased cellular respiration, deficits in energy production and increased ROS, leading to more dysfunction. The delivery of recombinant TFAM to normal mice increased their motor endurance and increased mitochondrial respiration [56]. Treatment of a mouse model of Parkinsonism with recombinant TFAM led to improved motor endurance and motor learning [57].

Similarly, a nuclear localization signal (NLS) was used in conjunction with a polyarginine CPP to direct nuclear targeting. Wang *et al*. used the well-studied NLS from simian virus 40 large-T antigen in combination with octa-arginine in order to increase DNA delivery to the nuclear compartment. The NLS-octa-arginine compound effectively delivered luciferase DNA to two different cell lines [58].

A cyclical new peptide, cyc 3, is able to show localization to the nucleolus. Cyc 3 is a cyclical CPP based on the cationic antimicrobial peptide (CAP18), which takes advantage of the recent observations that peptide rigidity plays a role in its uptake efficiency [59]. Upon incubation with cells at 4 °C, the peptide displayed a high degree of nucleolar targeting. Cyc3 was able to promote efficient transfection of cells with green fluorescent protein DNA, demonstrating a potential toward future therapeutic use [60].

### 4.3. Immune Response to Administration of Cell-Penetrating Peptides

CPP sequences are in many cases novel to the organism to which they are being administered and it is thus possible that they could elicit an immune response. It is therefore important to establish the potential immunogenic effects of CPP administration. In a study by Carter *et al.*, three of the most commonly used CPPs—Tat, penetratin and transportan—were examined for potential innate immunity induction. Epithelial cells from skin, lung and intestine, the cells most likely to first come into contact with systemically administered CPP therapeutics, were incubated with albumin conjugated to one of the three CPPs evaluated. Although the cells internalized the CPP and cargo, their viability was unaltered. Furthermore, they failed to produce an innate immune response as assessed by lack of activation of phosphorylated NF-κB, a signaling molecule downstream of toll-like receptors, and failure to secrete epithelial-specific interleukins, IL-6 and IL-8 [61].

The immunogenicity of Tat, transportan and some synthetic derivatives of transportan was also evaluated by Suhorutsenko *et al.* These peptides were incubated with human white blood cells either alone or with plasmid or siRNA. In all cases, no immune response was observed, as measured by cytokine release. These results were confirmed in mice and, similarly, there were no increases in cytokines detected in serum [62].

These studies showed that it is possible to deliver CPPs without eliciting an immune reaction; however, given the breadth and diversity of available CPPs, assessment of immunogenicity should be performed for each individual CPP. Additionally, coupling of a CPP with cargo can result in generation of novel epitopes such that even if an individual isolated CPP does not elicit an immune response, it may do so when conjugated to a particular cargo. Penetratin alone, for example, does not cause an immune reaction, but when coupled to an siRNA, the complex does elicit an innate immune response. The same siRNA complexed with Tat, however, did not elicit a response [63]. These data caution against drawing generic conclusions on the safety of CPPs based on studies with individual penetrating peptides and cargoes. It is clear that the immunogenicity of each CPP-cargo complex must be determined empirically.

### 4.4. Toxicity of CPP Administration

*In vitro* studies on the cytotoxicity of CPPs show that cationic CPPs are less toxic and can be tolerated by the cells at much higher concentrations than amphipathic CPPs, such as transportan and model amphipathic peptide (MAP). Commonly used assays, such as cell viability, proliferation and leakage of lactate dehydrogenase (LDH) demonstrate virtually no toxic effects of Tat and penetratin. In contrast, MAP and transportan show an effect on cell viability and membrane integrity at relatively low concentrations, such as those used for intracellular delivery [64,65]. Similar results are found with more novel methods for assessing the effect of CPPs on cell function. A comprehensive metabolomics analysis comparing the effect of five different CPPs confirmed the low impact of cationic peptides, while showing that transportan can cause oxidative stress [66]. As with immunogenicity, the toxicity profile of a CPP is heavily influenced by the nature of its associated cargo, as well as the attachment site of the cargo. In the case of transportan, orthogonal attachment of cargo was shown to be significantly less toxic than N-terminal attachment [65]. This observation could be explained by a location-dependent alteration in the hydrophobicity or amphipathicity of the peptide.

Although comprehensive *in vivo* toxicity studies for CPPs are generally not available, a small number of published animal studies as well as the approval of several CPP formulations for use in clinical trials attest to the general safety of therapeutic CPP molecules at the doses studied [67,68,69]. One exception is a recent *in vivo* study using intravenously delivered nona-arginine (r_9_), in which mice experienced immediate respiratory collapse and death [70]. At lower doses of peptide, however, mice did not develop observable long-term toxicity. In rats treated with twice-daily intranasal administration of insulin and penetratin for up to 30 days, no long-term toxicity was observed [71]. Histology studies of the nasal mucosa revealed healthy, intact membranes with no evidence of toxicity and no statistically significant evidence of increased LDH leakage. Interestingly, when the CPPs were administered alone, there was a trend toward less leakage as when compared to co-administration with insulin. These findings support the notion that the safety of each CPP used for therapy should be determined individually, in conjunction with its associated cargo [71].

As recently noted by Verdurmen and Brock, several compounds involving CPPs have been tested in humans with no serious adverse effects [67]. KAI-9803 is a δ protein kinase C (δ PKC) inhibitor that was shown to reduce ischemia/reperfusion-related injury after myocardial infarction in animal models [72]. A subsequent clinical trial testing the safety and efficacy of the compound in humans after intracoronary administration found that the drug has an overall good safety profile, with no significant differences in clinical laboratory values between placebo and study drug and no serious adverse events, although the study was not powered sufficiently to demonstrate efficacy [73]. These studies demonstrate that it is possible to safely administer CPP constructs *in vivo* so long as careful consideration is given to potentially novel effects of conjugating a CPP to a previously untested cargo.

## 5. Conclusions

Numerous studies using cell-penetrating peptides demonstrate the feasibility and efficacy of using CPP-tagged proteins as therapeutic agents in experimental systems. To the best of our knowledge, however, no CPP-tagged proteins have yet entered human clinical trials. Nevertheless, given the numerous CPP-peptides that have entered clinical trials, we are cautiously optimistic that CPP-proteins will eventually be useful therapeutic agents. Expanding the use of therapeutic proteins can address many unmet needs and has potential for higher therapeutic specificity with acceptable safety. In addition, CPPs can reduce the dosing necessary to achieve therapeutic levels and can be used to facilitate delivery to specialized cells, thus reducing overall toxicity. The ongoing discovery of numerous CPPs, the extensive use of CPP-protein therapy in experimental disease models and the fact that CPP-aided therapies have entered human clinical trials speaks to their potential impact on developing novel CPP-protein therapies for human disease.

## Abbreviations

CPP: cell-penetrating peptide
PTD: protein transduction domain
HIV: human immunodeficiency virus
Tat: trans-activator of transcription
ANTP: antennapedia
INF: influenza virus-derived peptide
GALA: glutamic acid-alanine-leucine-alanine
CTL: cytotoxic T-cell lymphocyte
TRP2: tyrosinase-related protein 2
scFv: single chain variable fragment
MDM2: murine double minute 2 homolog
CGD: chronic granulomatous disease
PHOX: phagocyte NADPH oxidase
ROS: reactive oxygen species
LAD: lipoamide dehydrogenase
NDUFAF4: NADH dehydrogenase complex I assembly factor
PDHC: pyruvate dehydrogenase complex
XLMTM: X-linked myotubular myopathy
LSD: lysosomal storage disease
Ndi1: NADH-quinone internal oxidoreductase
MPP: mitochondria-targeting peptide
Mtx: methotrexate
mtCPP: mitochondrial-targeting CPP
5-FAM: 5(6)-carboxyfluorescein
MTS: mitochondrial-targeting signal
TFAM: mitochondrial transcription factor A
NLS: nuclear-targeting signal
CAP18: cationic antimicrobial peptide
IP: intraperitoneal
MAP: model amphipathic peptide
LDH: lactate dehydrogenase
r_9_: nona-arginine
δ PKC: delta protein kinase C
